# Laboratory Test Surveillance following Acute Kidney Injury

**DOI:** 10.1371/journal.pone.0103746

**Published:** 2014-08-12

**Authors:** Michael E. Matheny, Josh F. Peterson, Svetlana K. Eden, Adriana M. Hung, Theodore Speroff, Khaled Abdel-Kader, Sharidan K. Parr, T. Alp Ikizler, Edward D. Siew

**Affiliations:** 1 Geriatrics Research Education & Clinical Center (GRECC), Tennessee Valley Healthcare System (TVHS), Veteran's Health Administration, Nashville, TN, United States of America; 2 Department of Biomedical Informatics, Vanderbilt University School of Medicine, Nashville, TN, United States of America; 3 Division of General Internal Medicine, Department of Medicine, Vanderbilt University School of Medicine, Nashville, TN, United States of America; 4 Department of Biostatistics, Vanderbilt University School of Medicine, Nashville, TN, United States of America; 5 Division of Nephrology, Department of Medicine, Vanderbilt University School of Medicine, Nashville, TN, United States of America; University of Leicester, United Kingdom

## Abstract

**Background:**

Patients with hospitalized acute kidney injury (AKI) are at increased risk for accelerated loss of kidney function, morbidity, and mortality. We sought to inform efforts at improving post-AKI outcomes by describing the receipt of renal-specific laboratory test surveillance among a large high-risk cohort.

**Methods:**

We acquired clinical data from the Electronic health record (EHR) of 5 Veterans Affairs (VA) hospitals to identify patients hospitalized with AKI from January 1^st^, 2002 to December 31^st^, 2009, and followed these patients for 1 year or until death, enrollment in palliative care, or improvement in renal function to estimated GFR (eGFR) ≥60 L/min/1.73 m^2^. Using demographic data, administrative codes, and laboratory test data, we evaluated the receipt and timing of outpatient testing for serum concentrations of creatinine and any as well as quantitative proteinuria recommended for CKD risk stratification. Additionally, we reported the rate of phosphorus and parathyroid hormone (PTH) monitoring recommended for chronic kidney disease (CKD) patients.

**Results:**

A total of 10,955 patients admitted with AKI were discharged with an eGFR<60 mL/min/1.73 m^2^. During outpatient follow-up at 90 and 365 days, respectively, creatinine was measured on 69% and 85% of patients, quantitative proteinuria was measured on 6% and 12% of patients, PTH or phosphorus was measured on 10% and 15% of patients.

**Conclusions:**

Measurement of creatinine was common among all patients following AKI. However, patients with AKI were infrequently monitored with assessments of quantitative proteinuria or mineral metabolism disorder, even for patients with baseline kidney disease.

## Introduction

Patients surviving AKI are at increased risk for long-term loss of kidney function and mortality [1], [2], [3], [4], [5], [6]. As AKI identifies both patients at risk for developing incident CKD along as well as acceleration of disease among those with prevalent chronic kidney disease (CKD) [7], [8], characterizing the patterns of care following AKI is an important first step to developing strategies that can potentially improve outcomes among AKI survivors. Kidney function and proteinuria may also predict recurrent AKI [9], a potentially important mechanism for potential disease progression following AKI [10].

Recently published guidelines by the Kidney Disease Improving Global Outcomes (KDIGO) panel recommend that patients who have experienced AKI be evaluated with a follow-up serum creatinine by 3 months to assess for resolution, new onset, or worsening of pre-existing CKD and to consider patients without CKD to be at ‘increased’ risk [11]. Current clinical practice guidelines for patients with CKD recommend they be appropriately monitored for disease progression (i.e. serum creatinine), the development of risk factors that associate with disease progression (i.e. proteinuria), and complications of kidney disease that may contribute to morbidity and mortality (e.g. disorders of mineral metabolism) [12]. The CKD guidelines state that dipstick screening for proteinuria among the general population is acceptable but advocate more quantitative and specific measurements including albumin-to-creatinine ratio (ACR) or protein-to-creatinine ratio (PCR) in patients deemed to be at ‘increased’ risk for progressive disease [13]. A summary of these recommendations is presented in Table 1 among patients with acute kidney injury, chronic kidney disease, and diabetes.

**Table 1:** Summary of KDIGO and American Diabetes Association (ADA) recommendations regarding surveillance among acute kidney injury, chronic kidney disease, and diabetic patient cohorts.

Clinical Setting	Laboratory Test(s)	Monitoring Recommendation
Acute Kidney Injury [47]	Serum Creatinine	3 months following AKI to assess for CKD:
		If CKD, use CKD monitoring recommendations
		If not CKD, consider as increased CKD risk
After Acute Kidney Injury without Chronic Kidney Disease [47]	Serum Creatinine, Any Urine Protein	Recommend undergoing testing to estimate renal function and markers of chronic kidney disease but do not specify an interval
Chronic Kidney Disease [15]	Serum Creatinine, Any Urine Protein, Urine Quantitative Protein	Every 12 months or every 4–6 months with GFR<30 or moderately increased proteinuria. Quantitative assessment preferred over dipstick urine protein.
Chronic Kidney Disease [15], [48]	Serum PTH, Serum Phosphate	Measurement at least once to establish baseline, with subsequent frequency of testing determined on an individual basis, with reasonable monitoring intervals every 12 months or every 3–6 months for GFR<30
Diabetes [49]	Urine Quantitative Protein	Every 12 months

We sought to determine the frequency of laboratory surveillance among survivors of AKI with evidence of impaired kidney function at the time of discharge. We hypothesized that AKI survivors would be infrequently assessed for kidney function recovery and proteinuria, and that patients with persistent impairment of kidney function would not be assessed for disorders of mineral metabolism (an early complication of chronic renal dysfunction) [14], [15]. We evaluated these hypotheses by examining the frequency and timing of measurement of serum creatinine, proteinuria, and serum phosphorous or intact parathyroid hormone (PTH) among AKI survivors within a regional Veterans Affairs (VA) Integrated Service Network (VISN 9) Health Care system.

## Materials and Methods

### Study Setting and Design

The study cohort pooled data from five Veterans Administration (VA) medical centers located in Nashville, TN, Murfreesboro, TN, Lexington, KY, Louisville, KY, and Huntington, WV. The VA is an integrated care network that includes acute inpatient hospitals, outpatient primary care and sub-specialty clinics, outpatient pharmacies, rehabilitation facilities, and long-term care facilities and domiciliaries. All VA clinical providers and allied health personnel are required to use the same electronic health record (EHR) for documentation and execution of all clinical care.

A retrospective cohort was collected of all adult (≥18 years) patients with a hospital admission complicated by AKI from January 1^st^, 2002 to December 31^st^, 2009. The Tennessee Valley Health System (TVHS) Veteran's Health Administration Institutional Review Board (IRB) and Research & Development Committees approved this study. This study was executed under expedited review with a waiver of HIPAA and informed consent approved by the IRB, written informed consent was not required. Because accurate date/time stamps were required for much of the work, full de-identification was not possible for the data, but removal of all patient names, social security numbers, and other identifiers except for dates of birth and death were performed in the initial data preparation step prior to analysis.

### Data Collection

All data were collected from the regional data warehouse, which included demographic information (e.g., age, gender, race, admitting service, and location), inpatient and outpatient procedure and diagnosis codes using CPT and ICD9 coding, chemistry and hematology laboratory data, fee basis records (non-VA care ordered by a VA provider), and inpatient and outpatient computerized provider order entry records.

Data are available through VA Informatics and Computing Infrastructure, which provides access to the VA electronic health record data for research purposes (http://www.hsrd.research.va.gov/for_researchers/vinci/). We are willing to share the SQL database extraction, transformation, and load scripts used to prepare the data and the R statistical code used in the analysis upon request to the corresponding author.

### Data Definitions

Acute kidney injury was defined as a 0.3 mg/dl or 50% increase in serum creatinine from the baseline serum creatinine to the peak inpatient serum creatinine, based on the Stage I definition of the Acute Kidney Injury Network (AKIN) creatinine criteria [16]. Based on our prior validation work, the baseline creatinine was defined as the mean observed outpatient serum creatinine value between 7–365 days prior to admission [17]. Estimated Glomerular Filtration Rate (eGFR) for all serum creatinine measures was calculated using the abbreviated Modification of Diet and Renal Disease (MDRD) equation as these were the values clinicians were provided [18], and CKD was defined as a baseline eGFR<60 mL/min/1.73 m^2^ [13].

Other chronic comorbidities (diabetes mellitus, hypertension, coronary artery disease, congestive heart failure, and peripheral arterial disease) were defined using administrative CPT and ICD-9 diagnostic codes collected from data prior to hospital admission, and detailed in Appendix S1. Extensive ICD-9 validation work has been performed previously in the Veterans Affairs [19] and in general populations [20]. Additional related ICD-9 codes used were aimed at increasing sensitivity to these validated codes.

### Cohort Inclusion/Exclusion Criteria

Patients were included if they had at least one outpatient serum creatinine measurement between 7 and 365 days prior to admission, at least one inpatient creatinine, and were hospitalized for greater than 24 hours [21]. For patients with multiple eligible AKI admissions, only the first qualifying admission was chosen. In order to select patients with continuity of care in the VA system beyond an isolated inpatient admission, patients were required to have encounters with the VA on two different dates within a year prior to the admission. An encounter was defined as any inpatient or outpatient diagnostic code, outpatient pharmacy fill record, laboratory test measurement, radiology test evaluation, pathology test measurement, or outpatient clinic visit within the VA or through VA fee basis care. Patients were excluded if they had end stage renal disease (ESRD) defined as receiving chronic dialysis therapy through ICD9 or CPT codes, a recorded dialysis procedure within 48 hours before admission, or had a baseline eGFR≤15 mL/min/1.73 m^2^. Patients with a history of renal transplant were also excluded. Patients still receiving dialysis therapy within 48 hours of discharge or receiving hospice/palliative care prior to discharge were excluded. Patient hospitalizations >30 days were also excluded because of systematic differences and complications in patients requiring care for long inpatient stays. Finally patients who died or experienced an improvement of renal function to ≥60 mL/min/1.73 m^2^ prior to discharge were excluded.

### Outcome Definitions

The primary outcomes were time from discharge to follow-up of serum creatinine, any proteinuria, quantitative proteinuria, and PTH or phosphorus as separate assessments. For any proteinuria, quantitative proteinuria, PTH, and phosphorus measurements, the times to improvement in kidney function, mortality, and hospice enrollment were included in the model as competing outcomes due to the lack of rationale for performing ongoing monitoring in those situations. Only mortality and hospice enrollment were modeled as competing risks for serum creatinine follow-up.

The definition of serum creatinine was all blood, serum, or plasma creatinine measurements performed. Urinary protein evaluation was separated into any proteinuria measurement and quantitative measurements restricted to spot microalbumin or albumin tests, protein to creatinine ratio tests, and timed urine collections for albumin and protein. The composite outcome of measurements of PTH or phosphorus included all such measurements in blood, serum, or plasma. Death was ascertained through an aggregate of administrative codes, the national death index, patient family reports, personnel direct family contact, and federal third party notifications.

### Statistical Analysis

Pre-admission patient demographics and chronic clinical conditions were summarized as medians and interquartile ranges (IQRs) for continuous variables and frequencies (%) for categorical variables. We used cumulative incidence functions (CIF) to estimate cumulative probabilities for occurrence of laboratory testing, improvement to eGFR≥60 mL/min/1.73 m^2^, and death or hospice care (treating these events as competing risks) [22], [23]. Because the occurrence of death, hospice or improvement of clinical condition preclude the need for monitoring of kidney injury, these outcomes are competing events effecting the proper estimation of incidence of surveillance using CIF whereas Kaplan-Meier function analysis would overestimate the probability of laboratory test surveillance. A competing risk analysis accounts for patient outcomes, as noted above, whose occurrence results in patients no longer being recommended to receive laboratory surveillance for proteinuria and mineral bone disease (MBD). Furthermore, reporting the cumulative probabilities of competing events that potentially explain non-receipt of care as well as the proportion of patients who “remain at risk” who have not yet received care may help provide some clinical context to facilitate interpretation of the true “gap” in care.

The following set of models measure the cumulative incidence at 3 months and 12 months for 1) laboratory testing surveillance occurring prior to renal function improvement, death, or hospice care, 2) renal function improvement without prior laboratory testing surveillance and prior to death or hospice care, and 3) death or hospice care without prior renal function improvement or surveillance testing. The models provide charting of surveillance incidence relative to the incidence of the competing factors and provide estimates of the proportion of surveillance testing and those remaining at risk at 3 months and 12 months post discharge. Patients who didn't have laboratory test evaluation, improvement to eGFR≥60 mL/min/1.73 m^2^, death or hospice care at the 12^th^ month of the surveillance period were censored. Aalen's variance estimator was used to estimate confidence intervals of cumulative probabilities [24] and confidence intervals for the proportion remaining at risk were calculated using the Greenwood method. In addition to the overall cohort analysis, stratified analyses of patients with a baseline eGFR≥60 mL/min/1.73 m^2^ versus <60 mL/min/1.73 m^2^, diabetes, or hypertension, were all separately performed. All analyses were performed using the R software for statistical computing, version 2.12.1 (http://www.r-project.org/).

## Results

### Subject Characteristics

**Figure 1:**
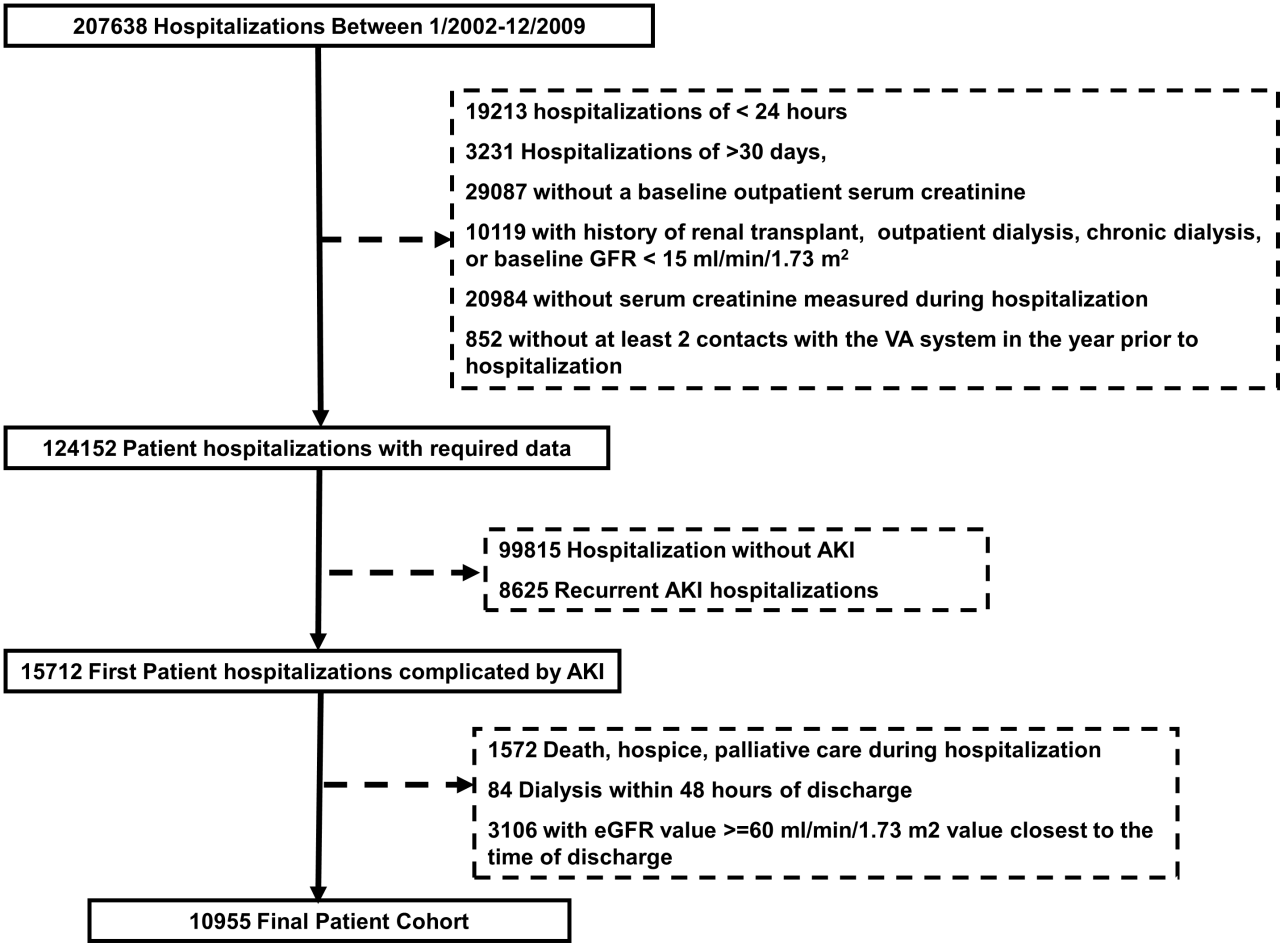
Study population flowchart. In each exclusion block, patients can appear in more than one exclusion criteria.

After applying inclusion and exclusion criteria (Figure 1), the study cohort included 10,955 patients with AKI during hospitalization who survived to discharge without hospice referral, dialysis-dependence, or an eGFR>60 mL/min/1.73 m^2^. Baseline cohort patient demographics and comorbidities prior to the index admission for the overall cohort and sub-cohorts with diabetes and hypertension are shown in Table 2. The median age of patients was 70 years (IQR: 60–78) with rates of chronic disease including diabetes mellitus of 57%, hypertension 88%, coronary artery disease 58%, congestive heart failure 34%, and peripheral vascular disease 21%. Of note, 5136 (47%) of patients had evidence of preadmission CKD defined by an outpatient baseline eGFR<60 mL/min/1.73 m^2^. The distribution of AKI severity during the index hospitalization using AKIN criteria included 8790 (80%) patients with AKIN stage I, 1063 (10%) with AKIN stage II, and 1099 (10%) with AKIN stage III injury.

**Table 2:** Demographics and Baseline Characteristics.

Variable	Full Cohort	Hypertension Sub-Cohort	Diabetes Sub-Cohort
Sample		9593	6196
Age	70 (60–78)	71 (61–79)	71 (61–79)
Men, n(%)	10667 (97%)	9375 (98%)	6066 (98%)
White, n(%)	9615 (88%)	8409 (88%)	5477 (88%)
Diabetes mellitus, n(%)	6196 (57%)	5806 (61%)	6196 (100%)
Hypertension, n(%)	9593 (88%)	9593 (100%)	5806 (94%)
Coronary artery disease, n(%)	6385 (58%)	5993 (62%)	4164 (67%)
Congestive heart failure, n(%)	3711 (34%)	3505 (37%)	2580 (42%)
Peripheral vascular disease, n(%)	2351 (21%)	2241 (23%)	1670 (27%)
Chronic Kidney Disease, n(%)			
CKD IIIa (eGFR 45–59 mL/min/1.73 m^2^)	2703 (25%)	2511 (26%)	1657 (27%)
CKD IIIb (eGFR 30–44 mL/min/1.73 m^2^)	1772 (16%)	1672 (17%)	1204 (19%)
CKD IV (eGFR 15–29 mL/min/1.73 m^2^)	661 (6%)	627 (7%)	449 (7%)
Median [IQR] Baseline Creatinine (mg/dl)	1.25 (1.02–1.58)	1.30 (1.05–1.60)	1.30 (1.07–1.66)
Median baseline estimated GFR, [IQR] mL/min/1.73 m^2^	62 (47–79)	60 (46–76)	58 (44–75)
Median Length of Stay, [IQR], Days	4.9 (2.8–8.6)	4.9 (2.8–8.3)	4.9 (2.8–8.6)

Continuous variables presented as median (interquartile range). GFR = glomerular filtration rate.

### Serum Creatinine Measurement

The cumulative incidence of serum creatinine testing adjusted for competitive risks within the full cohort was 68.7% (95% CI: 67.8–69.6) at 3 months and 85.3% (95% CI: 84.7–86.0) at 12 months. Only 4.9% of surviving patients did not have a creatinine measurement by the end of the follow-up period.

### Urinary Protein Measurement

**Figure 2:**
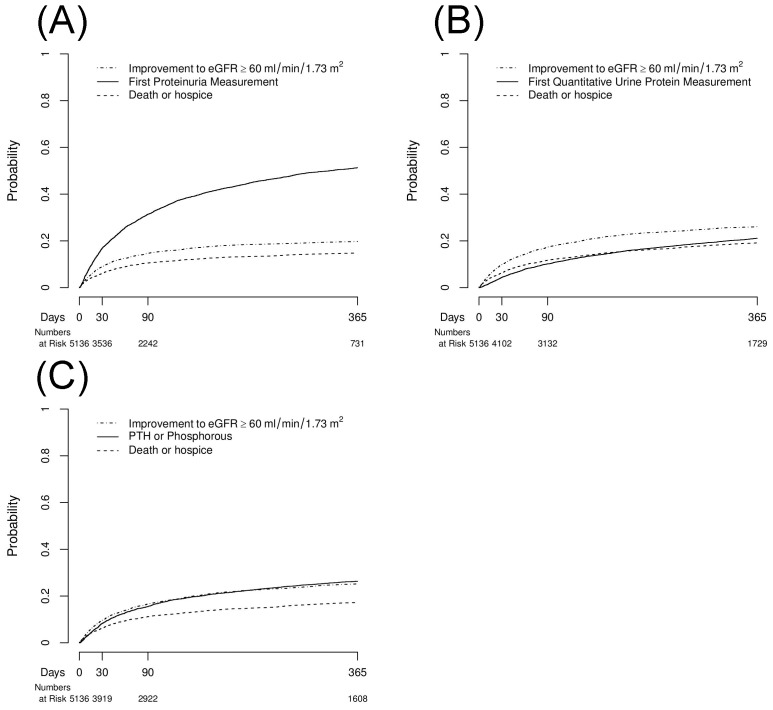
Cumulative Incidences of (A) Any Proteinuria Measurement, (B) Quantitative Proteinuria Measurement, and (C) PTH or Phosphorus Measurement Each Analyzed with Improvement in Kidney Function, and Death or Hospice as Competing Risks for one year following hospital discharge among Patients with Baseline eGFR<60 mL/min/1.73 m^2^.

**Figure 3:**
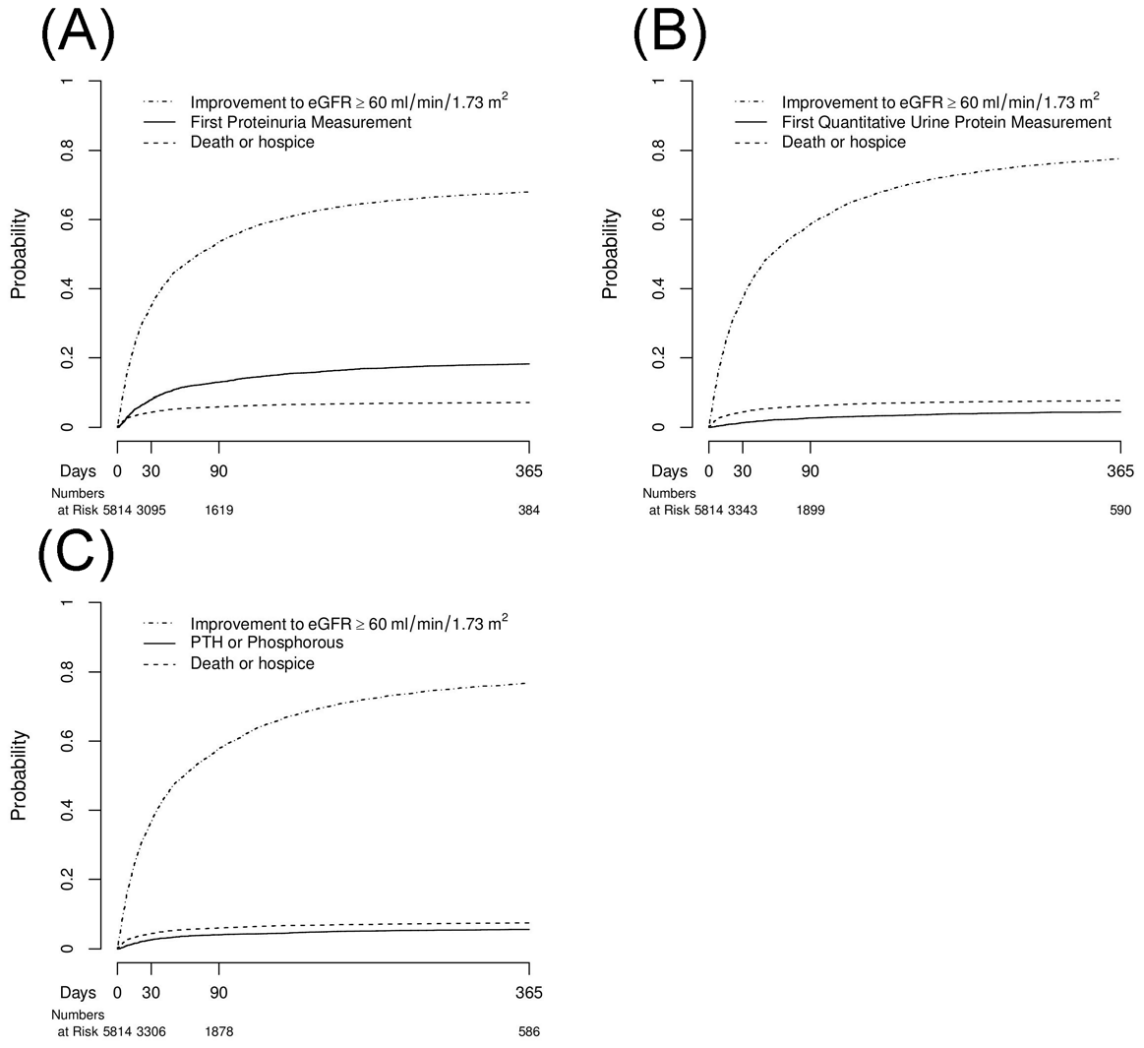
Cumulative Incidences of (A) Any Proteinuria Measurement, (B) Quantitative Proteinuria Measurement, and (C) PTH or Phosphorus Measurement Each Analyzed with Improvement in Kidney Function, and Death or Hospice as Competing Risks for one year following hospital discharge among Patients with Baseline eGFR≥60 mL/min/1.73 m^2^.

We measured the cumulative incidences of any dipstick or quantitative urinalysis measurement (Table 3, Figures 2b and 3b). Among the entire cohort, 33.7% underwent proteinuria testing measurement by 12 months prior to experiencing improvement of kidney function or death/hospice. A total of 10.2% of patients did not receive any proteinuria assessment at the end of follow-up. This number was 14.2% among patients with a baseline eGFR<60 ml/min/1.73 m^2^.

**Table 3:** (A) Urinary Proteinuria and (B) Quantitative Proteinuria each in Competing Incidence Functions with Improvement in Kidney Function (eGFR≥60 mL/min/1.73 m^2^) and Death or Hospice Care.

Event	Cohort	Baseline eGFR	3 months	12 months
(A) Any Proteinuria Measurement				
**Any Proteinuria Measurement**	All	≥60	13.0% (12.1–13.8)	18.2% (17.2–19.2)
	HTN	≥60	13.6% (12.6–14.5)	19.2% (18.1–20.3)
	DM	≥60	14.6% (13.3–15.9)	20.4% (19.0–21.9)
	All	<60	31.4% (30.2–32.7)	51.3% (49.9–52.6)
	HTN	<60	31.8% (30.5–33.1)	51.8% (50.3–53.2)
	DM	<60	33.8% (32.2–35.4)	55.0% (53.3–56.7)
**Improvement to eGFR**≥**60 mL/min/1.73 m^2^**	All	≥60	53.5% (52.2–54.8)	68.0% (66.8–69.2)
	HTN	≥60	53.3% (51.8–54.7)	67.8% (66.5–69.1)
	DM	≥60	54.2% (52.4–56.0)	67.7% (66.0–69.4)
	All	<60	14.7% (13.7–15.7)	19.7% (18.6–20.8)
	HTN	<60	14.6% (13.6–15.6)	19.7% (18.6–20.9)
	DM	<60	14.0% (12.8–15.2)	18.3% (17.0–19.7)
**Death or Hospice Care**	All	≥60	5.9% (5.4–6.6)	7.2% (6.5–7.9)
	HTN	≥60	5.7% (5.1–6.4)	7.0% (6.3–7.8)
	DM	≥60	5.6% (4.8–6.5)	7.0% (6.1–8.0)
	All	<60	10.5% (9.7–11.4)	14.8% (13.9–15.8)
	HTN	<60	10.0% (9.2–10.8)	14.3% (13.3–15.3)
	DM	<60	9.7% (8.7–10.7)	13.8% (12.6–15.0)
**Proportion Remaining at Risk**	All	≥60	27.8% (26.7–29.0)	6.6% (6.0–7.2)
	HTN	≥60	27.7% (26.5–39.0)	6.0% (5.3–6.7)
	DM	≥60	25.7% (24.1–27.3)	4.9% (4.1–5.6)
	All	<60	43.7% (42.3–45.0)	14.2% (13.3–15.2)
	HTN	<60	44.0% (42.5–45.4)	14.2% (13.3–15.2)
	DM	<60	42.8% (41.1–44.5)	12.9% (11.8–14.1)
(B) Quantitative Proteinuria Measurement				
**Quantitative Proteinuria Measurement**	All	≥60	2.7% (2.3–3.2)	4.5% (3.9–5.0)
	HTN	≥60	3.1% (2.6–3.6)	5.0% (4.4–5.7)
	DM	≥60	4.6% (3.9–5.4)	7.5% (6.6–8.5)
	All	<60	10.2% (9.4–11.0)	21.1% (20.0–22.3)
	HTN	<60	10.4% (9.6–11.3)	21.6% (20.5–22.8)
	DM	<60	12.6% (11.5–13.7)	26.7% (25.2–28.2)
**Improvement to eGFR**≥**60 ml/min/1.73 m^2^**	All	≥60	58.7% (57.4–59.9)	77.6% (76.6–78.7)
	HTN	≥60	58.4% (57.0–59.8)	77.8% (76.6–79.0)
	DM	≥60	59.3% (57.5–61.1)	77.1% (75.5–78.6)
	All	<60	17.3% (16.3–18.4)	26.1% (24.9–27.3)
	HTN	<60	17.2% (16.2–18.3)	25.9% (24.7–27.2)
	DM	<60	16.5% (15.3–17.8)	24.2% (22.8–25.7)
**Death or Hospice Care**	All	≥60	6.2% (5.6–6.8)	7.8% (7.1–8.5)
	HTN	≥60	5.9% (5.3–6.6)	7.6% (6.8–8.3)
	DM	≥60	5.9% (5.0–6.8)	7.7% (6.8–8.7)
	All	<60	11.7% (10.9–12.6)	19.2% (18.1–20.3)
	HTN	<60	11.2% 910.3–12.1)	18.7% (17.6–19.8)
	DM	<60	10.7% (9.6–11.7)	17.9% (16.6–19.2)
**Proportion Remaining at Risk**	All	≥60	32.7% (31.5–33.9)	10.1% (9.4–10.9)
	HTN	≥60	32.9% (31.5–34.2)	9.6% (8.8–10.5)
	DM	≥60	30.4% (28.7–32.1)	7.8% (6.8–8.7)
	All	<60	61.0% (59.6–62.3)	33.7% (32.4–35.0)
	HTN	<60	61.4% (60.0–62.8)	33.8% (32.5–35.2)
	DM	<60	60.6% (58.9–62.2)	31.2% (29.6–32.8)

The CIF was calculated in the entire cohort, among those with baseline eGFR≥60 mL/min/1.73 m^2^, and among those with baseline eGFR<60 mL/min/1.73 m^2^. Indicates the cumulative incidence probabilities and 95% confidence limits for each event and time interval during the surveillance period. HTN = Hypertension Sub-Cohort. DM = Diabetic Sub-Cohort.

We also measured the cumulative incidence of quantitative proteinuria (Table 3, Figures 2c, 3c). We found that 12.3% of the study cohort had quantitative proteinuria measurement prior to improving kidney function or experiencing death/hospice. Among survivors with persistently decreased eGFR<60 ml/min/1.73 m^2^, 21.2% of patients did not receive quantitative proteinuria assessment by the end of follow-up. This rate increased to 33.7% among survivors with a baseline eGFR<60 ml/min/1.73 m^2^.

### PTH or Phosphorus Measurement

Lastly, we found that 15.3% of patients had PTH or phosphorus measured by 12 months prior to improving kidney function (52.6%) or dying/receiving hospice care (12.1%) (Table 4, Figures 2d, 3d). Patients with baseline CKD had a higher likelihood of receiving PTH or phosphorus testing compared to those with normal pre-admission renal function (26.3% versus 5.6%). Among patients with pre-existing CKD, nearly one-third (31.3%) had neither a PTH nor phosphorus measured within 12 months.

**Table 4:** Cumulative Incidences for Receipt of PTH or Phosphorus, Improvement in Kidney Function (eGFR≥60 mL/min/1.73 m^2^), and Death or Hospice Care in the entire cohort, among those with baseline eGFR≥60 mL/min/1.73 m^2^, and among those with baseline eGFR<60 mL/min/1.73 m^2^.

Event		Baseline eGFR	3 months	12 months
**PTH or Phosphorus Measurement**	All	≥60	4.1% (3.6–4.6)	5.6% (5.1–6.2)
	HTN	≥60	4.0% (3.5–4.6)	5.5% (4.9–6.2)
	DM	≥60	3.9% (3.2–4.6)	5.5% (4.7–6.4)
	All	<60	15.5% (14.5–16.5)	26.3% (25.1–27.5)
	HTN	<60	16.0% (15.0–17.0)	27.0% (25.7–28.2)
	DM	<60	16.7% (15.4–18.0)	28.3% (26.8–30.0)
**Improvement to eGFR**≥**60 mL/min/1.73 m^2^**	All	≥60	57.8% (56.5–59.1)	76.8% (75.7–77.8)
	HTN	≥60	57.9% (56.5–59.3)	77.5% (76.3–78.6)
	DM	≥60	59.7% (57.9–61.4)	78.6% (77.1–80.1)
	All	<60	16.6% (15.6–17.7)	25.2% (24.0–26.4)
	HTN	<60	16.5% (15.5–17.6)	25.1% (23.8–26.3)
	DM	<60	15.9% (14.7–17.2)	24.0% (22.5–25.4)
**Death or Hospice Care**	All	≥60	6.0% (5.5–6.7)	7.5% (6.9–8.2)
	HTN	≥60	5.8% (5.2–6.5)	7.3% (6.6–8.1)
	DM	≥60	5.8% (5.0–6.7)	7.5% (6.6–8.5)
	All	<60	11.2% (10.4–12.1)	17.3% (16.2–18.3)
	HTN	<60	10.7% (9.8–11.6)	16.7% (15.6–17.7)
	DM	<60	10.2% (9.2–11.3)	16.2% (14.9–17.4)
**Proportion Remaining at Risk**	All	≥60	32.3% (31.1–33.5)	10.1% (9.3–10.9)
	HTN	≥60	32.6% (31.3–33.9)	9.7% (8.8–10.5)
	DM	≥60	30.8% (29.2–32.5)	8.4% (7.4–9.4)
	All	<60	56.9% (55.5–58.2)	31.3% (30.0–32.6)
	HTN	<60	57.1% (55.7–58.5)	31.4% (30.0–32.7)
	DM	<60	57.6% (55.9–59.2)	31.6% (30.0–33.2)

Indicates the cumulative incidence probabilities and 95% confidence limits for each event and time interval during the surveillance period.

### Summary of Results

**Figure 4:**
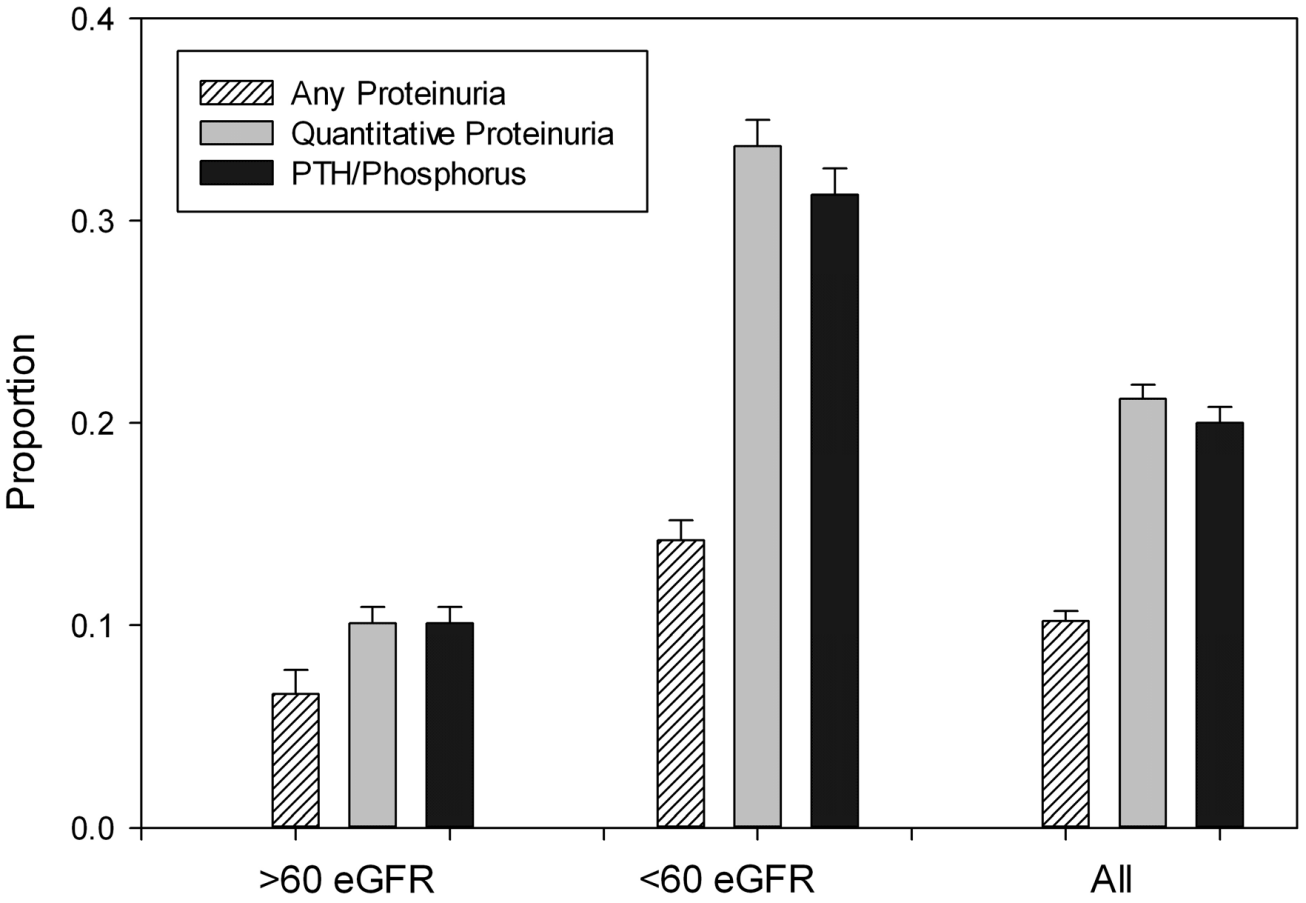
Proportion of patients Remaining at Risk (Still Eligible without Receipt) for Measurement of Any Proteinuria, Quantitative Proteinuria, or PTH or Phosphorus at one year post-discharge in the entire cohort, among those with baseline eGFR≥60 mL/min/1.73 m^2^, and among those with baseline eGFR<60 mL/min/1.73 m^2^. Indicates the cumulative incidence probabilities with competing risks from Improvement in Kidney Function (eGFR≥60 mL/min/1.73 m^2^) and Death or Hospice Care, and 95% confidence limits included.

Figure 4 is a summary of results summarizing the proportions of AKI patients who did not have any surveillance testing by 12 months adjusted for competing events.

### Sensitivity Analyses

In order to evaluate potential systematic differences for those patients with moderate to severe injury, we repeated our analysis defining AKI as only KDIGO Stage II–III injury (i.e. a minimum doubling of baseline serum creatinine or dialysis), and made them available in Appendix S2. The rates of improvement to eGFR≥60 mL/min/1.73 m^2^ were lower across all groups and strata at 30 days but were relatively similar at 365 days. Overall patterns of creatinine, any proteinuria, and quantitative proteinuria measurement were very similar, but PTH or phosphorus measurement increased from 15% to 24% in this population.

Because we found that a portion of those patients with baseline eGFR<60 mL/min/1.73 m^2^ experienced an eGFR≥60 mL/min/1.73 m^2^ at least once in the surveillance period following hospitalized AKI, we evaluated the eGFR values for patients with a baseline eGFR of <60 and 45–59 mL/min/1.73 m^2^, respectively, at the time of admission. We found that approximately 10% and 17% of these patients had an eGFR above 60 mL/min/1.73 m^2^ in the 180 days prior to admission, and approximately 12% and 20% of these patients experienced an eGFR above 60 mL/min/1.73 m^2^ in the year following the hospitalization with AKI.

## Discussion

In this study, we aimed to describe the rates of measurement of serum creatinine, proteinuria, and PTH and phosphorus among patients who survive AKI, an event associated with high risk for future loss of kidney function [8]. We found that serum creatinine and any proteinuria measurements were common following AKI. However, measurement of quantitative proteinuria and mineral metabolism among AKI survivors with persistent decreases in kidney function post-hospitalization were infrequently measured.

The most striking finding from this study are the paradoxically low rates of quantitative proteinuria assessments, especially among patients with pre-existing CKD. For example, among patients with baseline CKD who survived to 12 months, only 39% underwent quantitative proteinuria testing at 1 year. Sub-analyses among patients with diabetes and among patients with hypertension confirm the consistent pattern of low rates of assessment among each of those sub-groups with co-existing CKD. There was a small increase in surveillance among those patients with both diabetes and pre-existing CKD, but approximately a third of the patient population did not receive surveillance by one year. To emphasize the importance of this finding, both the CKD guidelines and the diabetes guidelines strongly recommend quantitative protein annually at a minimum.

In addition to being a key risk factor for CKD progression [25], [26], the presence of proteinuria is an important risk factor for AKI [7], [9], [27]. Accumulating data suggest that loss of kidney function in this population can often be non-linear and punctuated by recurrent episodes of AKI. [28], [29], [30] Consequently, monitoring for the development or worsening of proteinuria following an AKI event may be an important component of developing future risk reduction strategies. Because the longer term risks of AKI superimposed on CKD have only recently garnered increased attention, we suspect providers were unlikely to alter their established norms of care for CKD patients in our cohort.

While disorders of mineral metabolism have been observed during AKI [31], the extent to which AKI influences downstream derangements in mineral metabolism is unknown. KDOQI recommends initial laboratory assessment of PTH, calcium, phosphorus for all CKD patients followed by periodic surveillance depending on the severity and progression of CKD [30]. Although guidelines regarding management of mineral and bone disease are in need of higher quality evidence, we do not believe this justifies a nihilistic approach. Although changes in MBD markers are more prevalent in CKD Stage IV, approximately 10% of stable CKD stage 3 patients will develop hyperphosphatemia while 20–40% of stable CKD 3a–3b patients will develop secondary hyperparathyroidism [15]. Whether clinicians recommend dietary or pharmacologic interventions or watchful waiting while assessing for clinical manifestations, providers will require occasional lab testing to thoughtfully inform these decisions. Only about one quarter of patients with pre-existing CKD had PTH or phosphorous measured and over one-third were still eligible for surveillance by the end of the follow-up period. This is consistent with prior CKD literature, which has shown infrequent testing of phosphorus (26% to 68%) and PTH (3% to 38%), depending on the cohort and CKD severity [32], [33], [34]. The latter is particularly relevant given that patients with CKD who experience AKI are among those at the highest risk of experiencing accelerated decline to ESRD [35].

Early nephrology referral among both veteran and general populations with CKD has been shown to improve outcomes [36], [37], [38], [39], [40], but the rates of referral are low in both general outpatient settings and among those with recent hospitalized AKI [36], [41]. However, with recent data indicating continued rises in disease incidence [5], it is unlikely that the nephrology workforce will have the capacity to care for most of these patients. Consequently, the ability of primary caregivers to identify those at highest risk will be of increasing relevance to both streamline appropriate referral and targeting those for future interventions. Recent publications suggest that common lab values including serum phosphate and albuminuria can be used in predictive models to identify CKD patients at increased risk for end-stage renal disease [42] [43]. Similar work is needed in patients with AKI superimposed on CKD. As assessment for proteinuria and disorders of mineral metabolism are the precursors to effective management and potentially risk assessments, these results highlight the need to disseminate the components of nephrologist specialty care to primary care.

This study has a number of strengths. It is a multi-site study of a population of Veterans that leverages detailed laboratory and electronic health record data to establish rates of test measurement among a cohort with a high prevalence of CKD, diabetes, and hypertension. The study adds to the literature by providing estimates of surveillance among both the general high risk cohort of AKI survivors as well as the sub-group with pre-existing CKD. This study also included sensitivity analyses and evaluation for more severe degrees of injury. Stratification by baseline eGFR did show some threshold effects, particularly when the baseline was close to 60 mL/min/1.73 m^2^. However this reflects a clinically significant threshold for care, so we included analyses for the whole population and stratified by baseline eGFR to improved interpretation flexibility. In addition, severity of injury was noted to impact the rapidity but not the final rate of recovery and receipt of PTH and phosphorus measurement.

We recognize several study limitations. The veteran patient population may limit generalizability to other care settings. Excluding patients with lengths of stay greater than 30 days may potentially bias the population towards a healthier one, but this represents a very different sub-population within the VA, largely among patients extended rehabilitation, placement or psychosocial barriers to discharge, and for these reasons we excluded them. As noted above, we also did not evaluate the test result values in the follow-up period to determine whether they were abnormal or if clinical care changed in response to test ascertainment, and so a portion of those tested may not have received the recommended care following surveillance. While providers may have appropriately used ACEi or ARBs and optimally controlled BP without quantifying proteinuria, the literature on quality of CKD care suggests this is unlikely [44], [45], [46]. In addition, it is possible that some patients obtaining care at an outside healthcare facility were not captured. However, restriction of the cohort to those patients with frequent contacts with the VA likely limited this bias towards not receiving the recommended care, and rates of serum creatinine measurement were quite high, indicating that laboratory surveillance was conducted on the majority of these patients within the VA. Lastly, as optimal post-AKI management remains to be defined, it is yet unknown the extent that modifying risk factors such as proteinuria improve outcomes in this patient population and will likely require formal testing in future trials. However, we employed conservative criteria for appropriate laboratory surveillance, and in many patients, optimal surveillance is likely to consist of closer monitoring.

In summary, patients received high rates of serum creatinine surveillance following hospitalization complicated by acute kidney injury, but rates of monitoring for quantitative proteinuria, PTH, and phosphorus measurement were low among the patients, regardless of AKI severity. Clinicians delivering outpatient care to these patients following hospitalization, particularly among those patients with additional risk factors for worsening kidney disease, such as pre-existing CKD and diabetes, should ensure that patients receive the recommended laboratory test follow-up. These findings highlight opportunities to improve clinical care among AKI survivors receiving a majority of care outside of nephrology specialist care [41]. Opportunities that exist for improving proteinuria and CKD-MBD management include increasing the frequency of nephrology management for high risk patients and providing educational as well as clinical decision support tools to assist primary care providers.

## Supporting Information

Appendix S1 (DOCX)

Appendix S2 (DOCX)
