# Supplementary material for: Laboratory Test Surveillance following Acute Kidney Injury
Source: PLoS One. 2014 Aug 12;9(8):e103746. doi: 10.1371/journal.pone.0103746 (PMC4130516; doi:10.1371/journal.pone.0103746)
Supplement: Appendix S1 — (DOCX) [file pone.0103746.s001.docx]

**Appendix S1**

| Diagnosis | Definition |
| --- | --- |
| Hypertension | ICD-9 Outpatient or Inpatient Discharge Codes: 401*, 402*, 403*, 404*, and 405* |
| Diabetes Mellitus | ICD-9 Outpatient or Inpatient Discharge Codes: 250* (primary diabetes), 249* (secondary diabetes), 357.2* (neuropathy in diabetes), 362* (diabetic retinopathy), 366.41* (diabetic cataract), 648* (diabetes in pregnancy) |
| Coronary Artery Disease | ICD-9 Outpatient or Inpatient Discharge Codes: 440*, 410*, 411*, 413*, 414* |
| Peripheral Vascular Disease | ICD-9 Outpatient or Inpatient Discharge Codes: 443*, 440.2*, 440.3*, 440.4* |
| Congestive Heart Failure | ICD-9 Outpatient or Inpatient Discharge Codes: 428*, 425*, 398.91, 402.01, 402.11, 402.91, 404.91, 404.93 |
| Hospice | ICD-9 Outpatient or Inpatient Discharge Codes: V66.7*  Outpatient CPT Codes: 99377 |
| Renal transplant | ICD-9 Outpatient or Inpatient Discharge Codes: 55.6, 55.61, 55.69, 996.81, V42.0  ICD-9 Inpatient Procedure Codes: 00.91, 00.92, 00.93  Outpatient CPT Codes: 50380, 50365, 50360 |
| Dialysis | Inpatient ICD-9 Procedure Codes: 12.55, 39.95*, 54.98*  Outpatient and Fee Basis ICD-9 Diagnosis Codes: 585.6, V45.1, V56.0, V56.2, V56.3, V56.31, V56.32, V56.8, V58.8  Outpatient CPT Codes: 90935, 90937, 90947, 90989, 90993, 90921, 90925 |
