# Supplementary material for: Laboratory Test Surveillance following Acute Kidney Injury
Source: PLoS One. 2014 Aug 12;9(8):e103746. doi: 10.1371/journal.pone.0103746 (PMC4130516; doi:10.1371/journal.pone.0103746)
Supplement: Appendix S2 — (DOCX) [file pone.0103746.s002.docx]

**Appendix S2:** Sensitivity Analysis for the Outcome of AKIN Stage 2+ AKI.

*Table 1: Demographics and Baseline Characteristics. Continuous variables presented as median (interquartile range)*

| Variable | Value |
| --- | --- |
| Age | 68 (59-77) |
| Men, n(%) | 2491 (97%) |
| White, n(%) | 2165 (84%) |
| Diabetes mellitus,n(%) | 1473 (57%) |
| Hypertension,n(%) | 2263 (88%) |
| Coronary artery disease,n(%) | 1375 (54%) |
| Congestive heart failure,n(%) | 870 (34%) |
| Peripheral vascular disease,n(%) | 545 (21%) |
| Chronic Kidney Disease,n(%) |  |
| CKD IIIa (eGFR 45-59 ml/min/1.73 m^2^) | 448 (17%) |
| CKD IIIb (eGFR 30-44 ml/min/1.73 m^2^) | 357 (14%) |
| CKD IV (eGFR 15-29 ml/min/1.73 m^2^) | 478 (19%) |
| Median [IQR] Baseline Creatinine (mg/dl) | 1.3 (1.0-1.9) |
| Median baseline estimated glomerular filtration rate, [IQR] ml/min/1.73 m^2^ | 60 (38-82) |
| Median Length of Stay, [IQR], Days | 6.7 (3.8 – 11.8) |

**Table 2: (A) Any Urinary Proteinuria and (B) Quantitative Proteinuria each in Competing Incidence Functions with Improvement in Kidney Function (eGFR** ≥ **60 ml/min/1.73 m^2^) and Death or Hospice Care. The CIF was calculated in the entire cohort, among those with baseline eGFR** ≥ **60 ml/min/1.73 m^2^, and among those with baseline eGFR < 60 ml/min/1.73 m^2^.** Indicates the cumulative incidence probabilities and 95% confidence limits for each event and time interval during the surveillance period.

| **Event** | **Baseline**  **eGFR** | **3 months** | **12 months** |
| --- | --- | --- | --- |
| 1. Any Proteinuria Measurement | | | |
| **Any Proteinuria Measurement** | All | 27.0% (25.3-28.7) | 36.8% (34.9-38.7) |
|  | ≥60 | 16.1% 914.1-18.1) | 20.6% (18.4-22.8) |
|  | <60 | 38.0% (35.3-40.6) | 53.0% (50.2-55.7) |
| **Improvement to eGFR** ≥ **60 ml/min/1.73m^2^** | All | 31.9% (30.1-33.7) | 39.1% (37.2-40.9) |
|  | ≥60 | 52.5% (49.8-55.2) | 63.0% (60.3-65.6) |
|  | <60 | 11.3% (9.6-13.1) | 15.1% (13.2-17.1) |
| **Death or Hospice Care** | All | 12.5% (11.3-13.8) | 16.2% (14.8-17.6) |
|  | ≥60 | 9.6% (8.1-11.3) | 11.2% (9.6-13.0) |
|  | <60 | 15.4% (13.5-17.5) | 21.1% (18.9-23.4) |
| **Proportion Remaining at Risk** | All | 28.7% (27.0-30.5) | 8.0% (7.0-9.1) |
|  | ≥60 | 22.0% (19.7-24.2) | 5.2% (4.0-6.4) |
|  | <60 | 35.5% (32.8-38.1) | 10.8% (9.1-12.5) |
| 1. Quantitative Proteinuria Measurement | | | |
| **Quantitative Proteinuria Measurement** | All | 7.4% (6.5-8.5) | 12.4% (11.2-13.7) |
|  | ≥60 | 3.5% (2.6-4.6) | 5.0% (3.9-6.3) |
|  | <60 | 11.4% (9.7-13.2) | 19.8% (17.7-22.0) |
| **Improvement to eGFR** ≥ **60 ml/min/1.73m^2^** | All | 36.5% (34.6-38.3) | 47.0% (45.0-48.9) |
|  | ≥60 | 59.2% (56.4-61.8) | 73.7% (71.2-76.1) |
|  | <60 | 13.8% (12.0-15.8) | 20.2% (18.0 – 22.4) |
| **Death or Hospice Care** | All | 13.9% (12.6-15.2) | 19.6% (18.1-21.2) |
|  | ≥60 | 10.1% (8.5-11.8) | 12.1% (10.4-13.9) |
|  | <60 | 17.7% (15.7-19.8) | 27.2% (24.8-29.7) |
| **Proportion Remaining at Risk** | All | 42.4% (40.4-44.3) | 21.0% (19.4-22.6) |
|  | ≥60 | 27.4% (25.0-29.9) | 9.2% (7.6-10.8) |
|  | <60 | 57.3% (54-5-60.0) | 32.8% (30.2-35.4) |

**Table 3: Cumulative Incidences for Receipt of PTH or Phosphorus, Improvement in Kidney Function (eGFR** ≥ **60 ml/min/1.73 m^2^), and Death or Hospice Care in the entire cohort, among those with baseline eGFR** ≥ **60 ml/min/1.73 m^2^, and among those with baseline eGFR < 60 ml/min/1.73 m^2^.** Indicates the cumulative incidence probabilities and 95% confidence limits for each event and time interval during the surveillance period.

| **Event** | **Baseline**  **eGFR** | **3 months** | **12 months** |
| --- | --- | --- | --- |
| **PTH or Phosphorus**  **Measurement** | All | 10.8% (9.6-12.0) | 23.7% (22.1-25.4) |
|  | ≥60 | 4.9% (3.8-6.2) | 8.3% (6.9-9.9) |
|  | <60 | 16.6% (14.6-18.7) | 39.2% (36.5-41.8) |
| **Improvement to eGFR** ≥ **60 ml/min/1.73m^2^** | All | 22.8% (21.2-24.4) | 45.3% (43.4-47.2) |
|  | ≥60 | 30.0% (35.4-40.7) | 72.% (69.6-74.5) |
|  | <60 | 7.5% (6.1-9.0) | 18.6% (16.5-20.7) |
| **Death or Hospice Care** | All | 9% (7.9-10.2) | 17.0% (15.6-18.5) |
|  | ≥60 | 7.3% (6.0-8.8) | 11.4% (9.7-13.2) |
|  | <60 | 10.7% (9.1-12.4) | 22.6% (20.4-24.9) |
| **Proportion Remaining at Risk** | All | 58.2% (56.3-60.1) | 13.9% (12.6-15.3) |
|  | ≥60 | 50.6% (47.8-53.3) | 8.2% (6.7-9.7) |
|  | <60 | 65.9% (63.3-68.5) | 19.6% (17.5-21.8) |
